# Patient Preference for Pain Medication in the Emergency Department Is Associated with Non-fatal Overdose History

**DOI:** 10.5811/westjem.2018.4.37019

**Published:** 2018-06-11

**Authors:** Lauren K. Whiteside, Jason Goldstick, Aaron Dora-Laskey, Laura Thomas, Maureen Walton, Rebecca Cunningham, Amy S.B. Bohnert

**Affiliations:** * University of Washington, Department of Emergency Medicine, Seattle, Washington; † University of Michigan Medical School, University of Michigan Injury Center, Ann Arbor, Michigan; ‡ University of Michigan Medical School, Department of Emergency Medicine, Ann Arbor, Michigan; § University of Michigan Medical School, Addiction Center and Department of Psychiatry, Ann Arbor, Michigan; ¶ VA Center for Clinical Management Research, Department of Veterans Affairs Healthcare System, Ann Arbor, Michigan

## Abstract

**Introduction:**

Opioid overdose is a major public health problem. Emergency physicians need information to better assess a patient’s risk for overdose or opioid-related harms. The purpose of this study was to determine if patient-reported preference for specific pain medications was associated with a history of lifetime overdose among patients seeking care in the emergency department (ED).

**Methods:**

ED patients (18–60 years) completed a screening survey that included questions on overdose history, ED utilization, opioid misuse behaviors as measured by the Current Opioid Misuse Measure (COMM), and analgesic medication preferences for previous ED visits for pain with specific responses for preference for hydromorphone (Dilaudid®), morphine, ketorolac (Toradol®), “no preference” or “never visited the ED for pain.” We compared individuals who reported a lifetime history of overdose descriptively to those without a lifetime history of overdose. Logistic regression was used to determine factors associated with a history of overdose.

**Results:**

We included 2,233 adults in the analysis (71.5% response rate of patients approached) with 532 reporting at least one lifetime overdose. In the univariate analysis, medication preference was significantly associated with overdose history (p < .001); more patients in the overdose group reported preferring morphine and hydromorphone and those without a history of overdose were more likely to have no preference or say they had never visited the ED for pain. In the logistic regression analysis, patients with higher odds of overdose included those of Caucasian race, participants with a higher COMM score, preference for ketorolac, morphine or hydromorphone. Those who were younger, female and reported never having visited the ED for pain had lower odds of reporting a lifetime overdose. Having “any preference” corresponded to 48% higher odds of lifetime overdose.

**Conclusion:**

Patients with a pain medication preference have higher odds of having a lifetime overdose compared to patients without a specific pain medication preference, even after accounting for level of opioid misuse. This patient-reported preference could cue emergency physicians to identifying high-risk patients for overdose and other substance-related harms.

## INTRODUCTION

Overdose is a serious public health problem in the United States, where the rate of opioid-related deaths has increased 200% since 2000. 1 Additionally, non-fatal overdoses and hospitalizations for opioid overdose have increased significantly 2 and approximately half of all drug-related emergency department (ED) visits in 2011 involved misuse of a pharmaceutical or prescription drug (e.g., opioid, sedative, stimulant). 3 In an effort to curb this public health epidemic, there has been a concerted effort to promote safe opioid prescribing and limit opioids for chronic, non-cancer pain. Specifically, agencies such as the Centers for Disease Control and Prevention (CDC) have published guidelines for chronic opioid prescribing, 4 and the American College of Emergency Physicians has published an evidence-based clinical policy regarding issues around opioid prescribing from the ED. 5 However, many of these guidelines do not provide useful measures for identifying patients at risk for overdose or other opioid-related harms.

Emergency medicine providers are often charged with seeing patients with acute pain or acute exacerbations of chronic pain under hectic conditions without the benefit of an existing relationship or extensive information on the patient’s background. While there are many different self-report screening tools to understand individual patient risk for opioid-related harms when prescribing opioids, these tools are not routinely used in the ED. Many of these tools were developed specifically for patients initiating chronic opioids such as the Opioid Risk Tool, 6 making it difficult to interpret scores for patients in acute pain in the ED. State-based prescription drug monitoring programs (PDMP) that contain a registry of controlled substance prescribing linked to specific patients can be used to determine risk for diversion and has been shown to decrease prescribing of controlled substances. 7 However, the PDMP does not pick up on risky opioid behavior unrelated to diversion and doctor shopping and does not provide clinical outcomes regarding dispensed opioids such as previous overdose.

There has recently been much effort devoted to understanding patient-related risk factors associated with opioid-related harms including overdose. Patients on high daily opioid doses, concurrent use of sedative medications such as benzodiazepines, substance-use comorbidity, or patients who are prescribed extended-release or long-acting opioids are at increased risk for having an overdose. 8–12 Additionally, patients with a previous non-fatal overdose are at elevated risk for having subsequent overdose. 13–16 Many physicians use historical data from the electronic medical record (EMR) to review these treatment- and patient-related factors, including prior history of overdose or a history of polysubstance use.^10^,^14^ However, historical EMR data can offer an incomplete picture if patients seek care across different health systems or use different EDs.^17^

**Population Health Research Capsule**

What do we already know about this issue?

Overdose is a major public health

issue and emergency physicians don’t have good validated tools to assess risk for previous overdose.

What was the research question?

Is self-reported preference for pain medication during an emergency department (ED) visit associated with a previous overdose.

What was the major finding of the study?

ED patients with a self-report

preference for any pain medication have increased odds of previous overdose.

How does this improve population health?

Patient-reported preference for

pain medication should be explored further and could cue emergency physicians to identifying high-risk patients.

Information provided directly by patients could be a rapid and easy way to obtain useful information to supplement other tools such as the EMR or PDMP to understand opioid-related risks. Patients presenting for pain-related ED complaints may provide information regarding their preference for certain pain medications. Asking for a specific medication by name is one of the aberrant drug-related behaviors noted by pain medicine experts^18^ but this behavior has not been investigated in ED samples where patients often present for acute pain. To date, there have been no studies that seek to understand the association between a patient’s preference for a certain pain medication and risk for overdose. Researchers have theorized that ED patients’ chief complaints or requests for specific opioid medications may be predictive of non-therapeutic use,^19^ but there is little published data addressing these questions.

The objective of this study was to determine whether self-reported preference for pain medication during a visit to the ED was associated with a previous overdose among patients using the ED for care. We hypothesized that the odds of having a previous overdose will increase respectively as patients prefer hydromorphone (Dilaudid®), morphine, or ketorolac (Toradol®)compared to patients with no preference for a certain pain medication.

## METHODS

We conducted a secondary analysis of cross-sectional screening data obtained as part of the screening and recruitment phase of the Safety and Prevention Outcomes Study (SPOS), an ED-based, brief intervention aimed at reducing opioid overdose behavior in at-risk patients.^20^

### Study Setting and Population

We recruited participants from the University of Michigan Health System ED, a Level I trauma center located in Washtenaw County, MI, with a census of 85,000 adults annually. Participants were recruited Monday through Friday and two weekends per month between the hours of 6:30 am and 1:59 pm, from April 2013 to March 2014. Two trained research assistants (RAs) identified patients 18–60 years old without regard for chief complaint using computerized tracking logs, and they approached those placed in private treatment rooms. At the beginning of the shift, RAs would be randomly assigned areas of the ED to start recruitment and approach all potentially eligible patients in that zone who weren’t receiving medical care or talking with medical staff. Patients were excluded from screening if they did not understand English; were in police or corrections custody; had cognitive or other impairment precluding ability to consent (e.g., visual or hearing impaired); were medically unstable requiring immediate resuscitation (e.g., from major injury or sepsis), or were presenting for evaluation and treatment of sexual assault or suicidal ideation.

RAs obtained written informed consent and facilitated a brief, self-report screening survey using a tablet computer or pen-and-paper survey (when computers were unavailable), for which participants received a $1.00 gift. Participants were given the option to complete the screening independently using the tablet computer or by having the RA read the questions and input the answers (e.g., RA administered). During the consent process, we told possible participants we would be gathering information on physical and mental health and substance-use behaviors and depending on their answers they might be eligible for the next part. Participants who completed the screening did not know the intervention was to prevent overdose-risk behaviors. See figure for a more-detailed participant flowchart.

### Measurements

#### Outcome

The primary outcome measure was lifetime overdose, where overdose was defined as “*taking too much drugs or medications/pills and/or drinking too much alcohol,” i.e., “‘poisoning,’ ‘passing out,’ ‘nodding off,’ ‘blacking out,’ or an ‘overdose’ or ‘OD.*’” We designed this question to err on the side of high sensitivity, as we hypothesized that many patients in the ED would not identify these serious or life-threatening experiences with the term “overdose.” Lifetime overdose was determined by the answer to the question, “*How many times in your life has this kind of situation happened to you*?” Any answer other than “*never*” was coded as positive.

#### Demographics

Age, gender, race/ethnicity, employment status, annual income, and years of education were determined by self-report. Race/ethnicity categories included African-American, White, Hispanic/Latino, Asian, American Indian, and other, and were not mutually exclusive. Annual income was collapsed into four categories (≤ $19,999, $20,000–59,999, ≥ $60,000, or “Don’t know”), and employment was dichotomized as employed (full-time or part-time) or not employed (unemployed or retired). Years of education was categorized into three groups (high school or less, some college, graduated college).

#### ED Service Utilization and Visit Characteristics

ED utilization was determined by the self-reported number of ED or urgent care visits in the prior 12 months (including the baseline visit), and measured as 1, 2–3, 4–6, 7–9, 10–20, and ≥ 21. For this analysis, these scores were dichotomized as < 4 or ≥ 4. This cut-off was chosen to be consistent with previous definitions of “frequent utilizers” in the literature.^21^ Participants were queried as to whether they believed their current ED visit was related to “*drinking too much alcohol*,” “*taking too many medications*,” or “*taking too many substances*” (each reported as yes/no), and whether they had used opioid analgesics within the six-hour period prior to the current ED visit.

#### Analgesic Preferences and Opioid-misuse Behaviors

The key independent variable of interest was patient preference for analgesic medication, which was assessed by asking in the survey, “*When you visit the ER, what type of pain medication usually helps relieve your pain best?*” Patients were asked to choose among the following: hydromorphone, morphine, ketorolac, “no preference,” “I don’t know,” or “never visited ER for pain.” Patients who had no preference or reported “I don’t know” were combined into one category. We also included the level of non-medical use of prescription opioids (or “opioid misuse”) in the prior three months as measured by the sum of eight items (e.g., frequency of using prescription opioids not as prescribed) from the Current Opioid Misuse Measure (COMM). At the beginning of the COMM questions, participants were reminded that ‘*Pain medications also called “opioids” include Vicodin, codeine, Oxycontin, morphine, oxycodone, hydrocodone, methadone, hydromorphine, meperidine, fentanyl, or Norco, among others’* and then were able to provide answers to the eight COMM questions, which is a validated tool for assessing opioid misuse behaviors.^22^ Responses were measured on a 5-point scale (0: never, 1: rarely, 2: sometimes, 3: often, 4: very often); individuals’ scores were computed as the sum of these items to produce a 0–32 score. Patients responding with “No” to a lead-in question about previous non-medical use of prescription opioids received a total score of 0.

All analyses were performed using *R version 3.2.3* (R Core Team). We first calculated descriptive statistics among the total sample and among both those that did, and did not, have the outcome variable (lifetime history of overdose). We excluded from the sample participants with incomplete data for the outcome variable or the key independent variable of “analgesic preference.” Between-group comparisons of the distributions of each independent variable (e.g. univariate comparisons) were made using Kruskal-Wallis tests for quantitative variables and χ^2^ tests for categorical variables, respectively.

For adjusted comparisons, we used logistic regression. Variables included in the logistic model were selected based on clinical and theoretical considerations required to get properly adjusted estimates of the effect of patient analgesic preference. We preferred this approach to a stepwise selection procedure because such methods are known to produce anti-conservative inference (e.g., the resulting standard errors and *p*-values are biased low, and parameter estimates are biased high).^23^ To guard against separation issues in logistic regression, we ensured that there were at least 10 events per predictor variable included in the logistic regression. ED utilization was significant in the univariate analysis, but was not included in the adjusted model because this variable was hypothesized to be too similar to the preference variable, as participants who have a preference for pain medications were more likely to be exposed to previous ED visits for acute pain. Thus, the multivariable model included basic demographics (age, gender, race, education), opioids within six hours of ED visit, COMM score and preference for pain medication in the ED. Our first adjusted model provides separate adjusted odds ratios (aOR) for preference of ketorolac, morphine and hydromorphone. As a separate analysis, we also present an aOR combining these medication preferences into a variable labeled “any preference.” The included predictors were checked for collinearity and no variance inflation factors exceeded 1.8, indicating that the effect of collinearity was minimal. Individuals missing values on any of the included predictors (n=20) were excluded from this adjusted analysis for an analytic sample size of 2,213.

## RESULTS

A total of 3,146 eligible patients were approached, 2,249 completed the screen and 2,233 had complete data on the outcome variable as well as the analgesic preference variable and were included in this analysis (Figure).

In the univariate analysis, those with a lifetime overdose were older (p<0.001) and more likely to report being Caucasian (p<0.001) compared to participants who did not report a lifetime overdose; gender was not significant. (p<0.01). With regard to ED utilization, participants in the lifetime overdose group were more likely to have visited the ED at least four times in the prior year compared to those without a history of lifetime overdose (p < 0.001), and participants with a previous overdose were also more likely to report their current ED visit as related to too much alcohol or drugs (p < 0.001) and noted more pain medications and/or opioids within six hours of the ED visit (p<0.05); although overall frequency of these variables was low. Participants in the lifetime overdose group reported a mean COMM score of 2.6 (standard deviation [SD] 5.4) compared to those without a lifetime history of overdose, who reported a mean COMM score of 0.9 (SD 2.5) (p>0.001), indicating a higher level of current opioid misuse or nonmedical prescription opioid use among participants with a lifetime overdose. Likewise, more patients with a previous lifetime overdose had taken opioids (for appropriate medical purposes or inappropriate use) in the prior three months compared to those without a lifetime overdose (p <0.01). Overall, medication preference for acute pain varied across the two groups, with more participants in the lifetime overdose reporting preference for morphine (15.4% vs. 10.5%, p<0.01) and hydromorphone (16.0% vs. 8.2%, p<0.001) compared to those in the group without a history of lifetime overdose. There was no difference in the univariate analysis between groups for preference of ketorolac. Those without a lifetime overdose were more likely to report having never visited the ED for pain (p<0.001) or having no preference or an unknown preference (p<0.001) compared to those with a lifetime overdose (Table 1).

Nearly one-third of the sample (31%, n=532) reported a history of a lifetime overdose. Among those with overdose, the average number of previous overdoses was 3.1 (SD 1.91). In the logistic regression model evaluating factors associated with a lifetime overdose each analgesic medication (e.g. ketorolac, morphine, hydromorphone) was first included as a separate variable to understand how specific medication preferences were associated with a history of lifetime overdose (Table 2). In this adjusted model, demographic factors associated with a lifetime history of overdose include younger age (aOR [0.85]; 95% confidence interval [CI] [0.78–0.95]), female gender (aOR [0.80]; 95% CI [0.65–0.99]) and Caucasian race (aOR [2.03]; 95% CI [1.57–2.65]). COMM score indicating current opioid misuse was associated with increased odds of overdose (aOR 1.12 for each unit increase in COMM score, 95% CI [1.08–1.15]). Additionally, preference for hydromorphone at the current ED visit was associated with lifetime overdose (aOR [1.46], 95% CI [1.03–2.05]) as was preference for morphine (aOR [1.44], 95% CI [1.05–1.97]) and preference for ketorolac (aOR [1.62], 95% CI [1.01–2.57]). Participants who reported having never visited the ED for a pain-related complaint had a decreased odds of having a lifetime overdose compared to having visited an ED for pain but having no opioid preference (odds ratio [OR] [0.64]; 95% CI [0.48–0.86]).

To understand the association of lifetime overdose with any pain medication preference, we performed a second logistic regression model predicting lifetime overdose but with the medication-preference variables (e.g. hydromorphone, morphine, ketorolac) combined into one category to understand if “any preference” provided similar results. Interestingly, “any medication preference” had increased odds of lifetime overdose (OR [1.48], 95% CI [1.16–1.89]) suggesting that preference for any pain medication is associated with a history of overdose (Table 3); the OR for all other predictors remained virtually unchanged. Both models produced non-significant Hosmer-Lemeshow test results (for Table 2 and for Table 3), indicating no serious lack-of-fit in either model.

## DISCUSSION

Preference for pain medication type has not been examined previously as a predictor of lifetime overdose. In this investigation, patients’ specific preference for hydromorphone, morphine and ketorolac were all associated with a history of previous overdose after accounting for patient characteristics, including level of opioid misuse with ORs suggesting a modest but significant association. The overall conclusions did not change when ketorolac, morphine and hydromorphone preferences were combined, suggesting that any specific preference for pain medication is associated with a lifetime history of overdose.

Previous research has shown that patients who get opioids from the ED are at risk for opioid-related problems and those who misuse prescription opioids have elevated rates of ED utilization. Specifically, adolescent patients who receive parenteral opioids in the ED are more likely to report non-medical use of prescription opioids,^24^ and patients who receive opioids at discharge from the ED are at increased risk for long-term opioid use.^25^,^26^ Likewise, adults who reported using prescription opioids non-medically are more likely to have visited the ED compared to those who don’t report non-medical prescription opioid use.^27^ Preference for ketorolac was also independently associated with increased risk of previous overdose in the adjusted analysis suggesting that any pain medication preference is an important risk factor and not just preference for opioids.

Recent studies have shown that previous overdose history is an important predictor of future overdose risk.^14^–^16^ Approximately one in three participants (31%) in this study noted a lifetime history of overdose. The definition of overdose used for this study was intentionally broad, allowing for capture of adverse events associated with drug or alcohol use. A recent study by Bohnert and colleagues used a similar definition of overdose in a more urban ED population, which sampled patients presenting in the evening hours and found a prevalence of 12.1%,^28^ which is consistent with demographic trends for overdose. It is also important to note that alcohol contributes to overdose experiences. Banta-Green and colleagues reported on an opioid overdose intervention trial that recruited adult patients from an urban ED at elevated risk for opioid overdose, in which one-third of the participants reported they used alcohol when using prescription or illicit opioids.^29^ Overdose screening and intervention efforts from the ED have recently focused on opioids;^29^,^30^ however, alcohol may contribute to more severe overdose experiences, and patients using alcohol to the point of overdose either alone or in combination with other substances should be identified and receive education and intervention.

Using patient-reported preference for a specific pain medication as a marker or tool to identify elevated risk for previous overdose could allow for further targeted screening of patients at elevated risk for future opioid-related harms. These results suggest that information about analgesic preference that is commonly volunteered by patients during an ED visit for pain may have utility in informing an assessment and could be a way to identify patients who would benefit from additional screening or intervention to prevent misuse and/or overdose.

Previous investigators have shown that it is feasible to administer a self-report screening tool for opioid misuse at the time of discharge from the ED, which could help guide opioid prescribing.^31^ In this analysis, an increasing score based on eight items from the COMM a self-reported survey assessment, was associated with a lifetime history of overdose. Currently, there are no validated, self-report measures for opioid misuse in ED populations, and like other self-report tools the COMM has previously been used and validated in non-ED settings.^32^,^33^ Unlike other self-report screening tools for opioid misus e^34^ the COMM is relatively short and could easily be administered to patients in the ED.^35^ The high prevalence of endorsement of the COMM items suggests there was not a social desirability bias in answering these questions in the context of survey research when confidentiality is maintained and questions are answered privately using a tablet computer. In the logistic regression analysis, patient preference for pain medication still predicted increased odds of lifetime overdose after adjusting for COMM score, suggesting that preference for pain medication provides additional information about overdose compared to using only the COMM score alone. Importantly, the COMM and the self-reported medication preference provide information about potential opioid-related harms and both measures could be used in tandem as part of a way to understand individual risk for lifetime overdose. For example, clinicians could be cued in to pursuing validated, self-report screens such as the COMM in patients who self-report a pain medication preference to identify patients at risk for prescription opioid harms.

Interestingly, patients who had never been to the ED with a pain-related complaint had decreased odds of having a lifetime overdose. This is consistent with prior literature, which notes that patients on chronic opioid therapy have increased use of healthcare services including the ED.^35^ Also, patients without a prior visit to the ED for pain are less likely to be exposed to opioids. This suggests that prudent opioid prescribing at the point of an ED visit is important toward the goal of curbing the epidemic of overdose.

## LIMITATIONS

While this study provides novel information around correlations of previous overdose for patients currently in the ED, there are some important limitations to note. This is a cross-sectional analysis that supports an association between lifetime overdose and self-reported preference for pain medication but does not support a causal conclusion. Clinical characteristics including the reason for ED presentation was not obtained, although the screening strategy was broad and systematic. Patients were recruited during day-shift hours and we did not collect data during evenings or nights. Future studies should account for possible differences in patient presentation during evenings and overnight hours.

Consistent with all other self-report opioid-misuse measures, the COMM has not been validated in ED settings. The definition of overdose was intentionally broad. While this could be viewed as limiting generalizability, it also likely captures a wide range of overdose behaviors that wouldn’t be captured by a definition that was narrower in scope. The SPOS study occurred at a single institution and thus may not be generalizable to other settings such as rural EDs or to patients with different sociodemographic characteristics. Many measures were obtained through self-report including the main outcome of lifetime overdose. The survey question that provided the main exposure of interest (patient preference) was not cognitively tested with a similar population prior to this study.^36^ While this could be viewed as a limitation, there are several studies documenting the reliability and validity of self-report for risk behaviors using similar methods when privacy and confidentiality are protected,^37^–^39^ albeit not in the ED setting.

## CONCLUSION

ED patients with a preference for a specific pain medication have higher odds of having a lifetime overdose compared to patients without a specific pain-medication preference, above the association-attributed current opioid misuse. To our knowledge, this is the first such study to examine and find this association. Further study is needed to determine if patient preference for specific pain medication would lead to a prospective risk of overdose or other opioid-related problems. Emergency medicine providers should be cued to this patient-reported preference, which could assist in further understanding risk for overdose and other opioid-related harms.

## Figures and Tables

**Figure:**
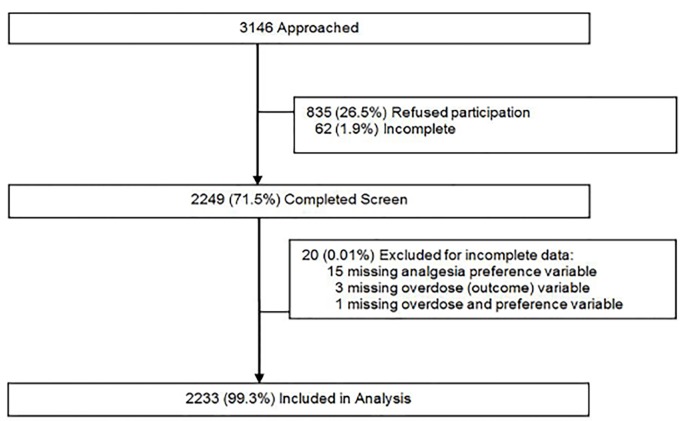
Study participation flowchart.

**Table 1:** Demographics, emergency department (ED) utilization and opioid use and experiences among ED patients.

Variable	Total (n=2233)	No previous lifetime overdose (n=1701)	Previous lifetime overdose (n=532)
Demographics			
Age***^a^		38.0 (12.8)	35.6 (12.4)
Female	(n=2233)	1074 (63.1%)	313 (58.8%)
Race***			
Caucasian***	1677 (75.1%)	1238 (72.8%)	439 (82.5%)
African American**	421 (18.9%)	346 (20.3%)	75 (14.1%)
Other (includes Asian and American Indian)	133 (5.9%)	107 (6.3%)	26 (4.9%)
Hispanic/Latino ethnicity	90 (4.0%)	75 (4.4%)	15 (2.8%)
Currently employed***	1451 (65.0%)	1146 (67.4%)	305 (57.3%)
Income*^b^			
≤19,999**	634 (28.4%)	454 (26.7%)	180 (33.8%)
$20,000–$59,000	662 (29.6%)	514 (30.2%)	148 (27.8%)
≥ $60,000	736 (33.0%)	577 (33.9%)	159 (29.9%)
Don’t know	181 (8.1%)	138 (8.2%)	43 (8.1%)
Schooling completed^c^			
Completed high school or less	512 (22.9%)	386 (22.7%)	126 (23.7%)
Completed some college	868 (38.9%)	652 (38.3%)	216 (40.6%)
Graduated college	515 (23.1%)	394 (23.2%)	121 (22.7%)
Emergency department (ED) utilization			
≥4 or more ED visits in the past year***^d^	421 (18.9%)	279 (16.4%)	142 (26.7%);
ED visit today due to alcohol or too many substances***	40 (1.8%)	13 (0.8%)	27 (5.1%)
Pain meds/opioids within 6 hours of ED visit*^e^	233 (10.4%)	165 (9.7%)	68 (12.8%)
Overdose experience			
Previous overdose in the last year***	196 (8.8%)	0 (0.0%)	196 (36.8%)
Current opioid misuse			
Taken any opioids in the past 3 months (yes)**^e^	794 (35.6%)	573 (33.7%)	221 (41.5%)
COMM score (mean)***^f^	1.3 (3.4)	0.9 (2.5)	2.6 (5.4)
Medication preference in the ED**			
No preference or don’t know***	1178 (52.8%)	915 (53.8%)	263 (49.4%)
Ketorolac	93 (4.2%)	63 (3.7%)	30 (5.6%)
Morphine**	260 (11.6%)	178 (10.5%)	82 (15.4%)
Hydromorphone***	225 (10.1%)	140 (8.2%)	85 (16.0%)
Never visited***	477 (21.4%)	405 (23.8%)	72 (13.5%)

*COMM*, Current Opioid Misuse Measure.

* p < 0.05,

** p < 0.001,

*** p≤ 0.0001 for univariate comparisons of “no overdose” compared to “overdose” groups.

a n=7 missing,

b n=20 missing,

c n= 5 missing,

d n=6 missing,

e n=1 missing,

f n=9 missing.

**Table 2:** Logistic regression predicting lifetime overdose with medication-preference variables separate.

Variable	Lifetime overdose with medication preference variables combined odds ratio (95% CI)
Demographics	
Age	0.85 (0.78, 0.92)***
Female	0.80 (0.65, 0.99)*
Caucasian race	2.03 (1.57, 2.65)***
High school or less	[ref]
College graduate	1.11 (0.84, 1.47)
Some college	1.04 (0.79, 1.36)
ED Utilization	
Opioid within 6 hrs of ED visit	0.83 (0.59, 1.17)
Current opioid misuse	
COMM score	1.12 (1.08, 1.15)***
Medication preference in the ED	
No preference	[ref]
Hydromorphone preference	n/a
Morphine preference	n/a
Ketorolac preference	n/a
Any preference	1.48 (1.16, 1.89)**
Never visited	0.64 (0.48, 0.86)**

*CI*, confidence interval; *ED*, emergency department; *COMM*, Current Opioid Misuse Measure.

* p < .05

** p < .01

*** p < .001

**Table 3:** Logistic regression predicting lifetime overdose with medication-preference variables combined.

Variable	Lifetime overdose with medication preference variables separate odds ratio (95% CI)
Demographics	
Age	0.85 (0.78, 0.92)***
Female	0.80 (0.65, 0.99)*
Caucasian race	2.03 (1.57, 2.65)***
High school or less	[ref]
College graduate	1.11 (0.84, 1.47)
Some college	1.04 (0.79, 1.36)
ED Utilization	
Opioid within 6 hrs of ED visit	0.83 (0.59, 1.17)
Current opioid misuse	
COMM score	1.12 (1.08, 1.15)***
Medication preference in the ED	
No preference	[ref]
Hydromorphone preference	1.46 (1.03, 2.05)*
Morphine preference	1.44 (1.05, 1.97)*
Ketorolac preference	1.62 (1.01, 2.57)*
Any preference	n/a
Never visited	0.64 (0.48, 0.86)**

*CI*, confidence interval; *ED*, emergency department; *COMM*, Current Opioid Misuse Measure.

* p < .05,

** p < .01,

*** p < .001
